# Attention and Executive Functions Are the Most Vulnerable Cognitive Domains in Patients With Drug‐Resistant Epilepsy Prior to Neurostimulation Therapy

**DOI:** 10.1002/brb3.71318

**Published:** 2026-03-31

**Authors:** Niina Lähde, Pabitra Basnyat, Hanna Lehtinen, Eija Rosti‐Otajärvi, Leena Kämppi, Jukka Peltola

**Affiliations:** ^1^ Faculty of Medicine and Health Technology Tampere University Tampere Finland; ^2^ Department of Neurology Tampere University Hospital Tampere Finland; ^3^ Department of Rehabilitation and Psychosocial Support Tampere University Hospital Tampere Finland; ^4^ Epilepsia Helsinki, Member of European Reference Network EpiCARE, Department of Neurology Helsinki University Hospital and University of Helsinki Helsinki Finland

**Keywords:** attention and executive functions, cognition, deep brain stimulation, drug‐resistant epilepsy (DRE), vagus nerve stimulation

## Abstract

**Background:**

Cognitive impairments are prevalent among patients with drug‐resistant epilepsy (DRE) referred for neurostimulation therapies. However, the performance characteristics across various cognitive domains in this patient group are not well established.

**Objective:**

To analyze the characteristics and severity of cognitive impairment in patients with DRE referred for neurostimulation therapy, specifically vagus nerve stimulation (VNS) or deep brain stimulation of the anterior nucleus of the thalamus (ANT‐DBS).

**Methods:**

The study included 53 consecutive patients with DRE who were considered for either VNS or ANT‐DBS therapy. As part of the preimplantation clinical protocol, all patients underwent neuropsychological evaluation. Cognitive tests were categorized into four domains: (1) attention and executive functions (AEFs), (2) memory and learning, (3) language functions, and (4) visual functions. Test performance was compared against established normative data.

**Results:**

More than one‐third of the patients exhibited cognitive impairment across all four assessed domains. Deficits in AEFs were the most prevalent and severe, affecting 66% of the cohort. These impairments were especially pronounced in patients treated with topiramate or zonisamide, as well as in those referred for ANT‐DBS.

**Conclusions:**

The presurgical neuropsychological evaluation of patients with DRE considered for neurostimulation therapy should place particular emphasis on AEFs. This focus provides a critical baseline for follow‐up and enables targeted monitoring of cognitive outcomes during neurostimulation treatment.

## Graphical abstract

More than one‐third of the patients with drug‐resistant epilepsy (DRE) referred for neurostimulation therapies exhibited cognitive impairment across all four assessed domains. Deficits in attention and executive functions were the most prevalent and severe, affecting 66% of the cohort. The presurgical evaluation of patients with DRE considered for neurostimulation therapy should include a comprehensive neuropsychological assessment, with particular emphasis on attention and executive functions. This focus provides a critical baseline for follow‐up and enables targeted monitoring of cognitive outcomes during neurostimulation treatment.

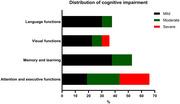

## 1 Introduction

Drug‐resistant epilepsy (DRE) is characterized by the persistence of seizures despite the use of two appropriate anti‐seizure medications (ASMs) at optimal doses, as defined by the International League Against Epilepsy (ILAE) (Kwan et al. 2010). Approximately 30% of patients with epilepsy develop DRE (Kwan and Brodie 2000). For most individuals with DRE, additional medication trials do not lead to seizure freedom, necessitating alternative treatment approaches (Chen et al. 2018). These alternatives include resective surgery and neurostimulation. Resective surgery is feasible for patients with a clearly identifiable seizure focus, provided its removal does not result in neurological or cognitive deficits (Wiebe and Jetté 2012). For patients who are not suitable candidates for surgery or do not benefit from it, neurostimulation offers a viable treatment option (Ryvlin et al. 2021; Touma et al. 2022). Currently, three neurostimulation therapies are approved for DRE: vagus nerve stimulation (VNS), deep brain stimulation of the anterior nucleus of the thalamus (ANT‐DBS), and closed‐loop responsive neurostimulation (RNS, available in the USA).

Cognitive dysfunction is a common comorbidity in DRE, affecting approximately 70%–80% of patients (Helmstaedter and Witt 2012; Lähde et al. 2021). These cognitive impairments are typically multifactorial in origin, involving both static factors such as the etiology and type of epilepsy and dynamic factors, including seizure frequency and severity, interictal epileptiform discharges, side effects of ASMs, and psychiatric comorbidities (Elger et al. 2004; Peltola et al. 2024). Additionally, clinical and demographic characteristics, including age at epilepsy onset, duration of epilepsy, and educational background, may also influence cognitive outcomes (Elger et al. 2004; Helmstaedter and Witt 2012; Kwan and Brodie 2001).

Patients with DRE being evaluated for neurostimulation therapies frequently exhibit executive dysfunction (Lähde et al. 2021). Notably, both VNS and ANT‐DBS have demonstrated positive effects on executive functions (Chan et al. 2018). Given that executive dysfunction reflects underlying brain connectivity, it may play a critical role in the outcomes of neurostimulation therapy (Järvenpää et al. 2018). However, there is a lack of clinical studies exploring executive functions in relation to other cognitive domains, such as memory and learning (ML), visual functions (VFs), and language functions (LFs), in patients with DRE undergoing evaluation for neurostimulation treatment. Therefore, this study aimed to evaluate the cognitive performance of patients with DRE who are being assessed for neurostimulation therapy with either VNS or ANT‐DBS.

## 2 Methods

### 2.1 Study Design

This was a non‐interventional study conducted at Tampere University Hospital, involving patients with DRE who were referred for evaluation of neurostimulation therapy. Data were collected prospectively as part of the clinical neurostimulation protocol and analyzed retrospectively. The study received approval from the Tampere University Hospital Development and Innovation Centre. Due to the registry‐based nature of the data, informed consent and ethical review board approval were not required, in accordance with Finnish research legislation. This manuscript complies with the Strengthening the Reporting of Observational Studies in Epidemiology (STROBE) guidelines.

### 2.2 Patients

The study population comprised 53 consecutive adult patients with DRE who were considered for neurostimulation treatment with VNS or ANT‐DBS. Patient selection for DBS or VNS was based on the need for improved seizure control in individuals with drug‐resistant focal epilepsy, rather than on specific clinical features such as epilepsy type. Patient preference was also taken into account. In patients with generalized epilepsy, the choice was VNS. All included patients were either unsuitable candidates for resective surgery, had declined the procedure, or had undergone resective surgery without benefiting from it. Prior to the decision to proceed with neurostimulation, the suitability for surgical intervention was assessed using video‐electroencephalography (EEG) telemetry, 18‐F‐fluorodeoxyglucose positron emission tomography (FDG‐PET), 3T magnetic resonance imaging (MRI), and, for some patients, additional studies such as stereo‐electroencephalography (SEEG), ictal single‐photon emission computed tomography (SPECT), and magnetoencephalography (MEG) were performed. Each patient underwent a neuropsychological evaluation. Patients with intellectual disability, visual impairments, or motor dysfunction were excluded.

### 2.3 Clinical Characteristics

Data on patient demographics at the time of neuropsychological evaluation were collected (Table 1). Patients were classified into three groups based on their years of formal education: 9 years, 10–12 years, and more than 12 years. Similarly, epilepsy duration was categorized into three groups: less than 10 years, 10–20 years, and more than 20 years. Epilepsy type was classified based on the location of the seizure onset zone into temporal lobe epilepsy (TLE), extra‐temporal epilepsy (XLE), and multifocal epilepsy. The etiology of epilepsy was determined using MRI findings and patients’ medical histories.

**TABLE 1:** Demographic and clinical characteristics of the study population.

Characteristics	All patients (*n* = 53)	VNS (*n* = 28)	ANT‐DBS (*n* = 25)	*p* value
**Age at baseline** Median (range) years	32 (19–70)	36.5 (19–70)	31 (19–56)	0.235^a^
**Sex; *n* (%)**				0.284^b^
Female	21 (39.6)	13 (46.4)	8 (32.0)	
Male	32 (60.4)	15 (53.6)	17 (68.0)	
**Years of education** Median (range)	12 (9–16)	12 (9–16)	12 (9–16)	0.088^a^
**Duration of epilepsy** Median (range) years	16.5 (1–49)	15 (2–41)	19 (1–49)	0.539^a^
**Epilepsy types; *n* (%)**				0.224^b^
TLE	24 (45.3)	15 (53.6)	9 (36.0)	
XLE	19 (35.8)	10 (35.7)	9 (36.0)	
Multifocal	10 (18.9)	3 (10.7)	7 (28.0)	
**ILAE etiology; *n* (%)**				0.812^b^
Structural	24^c^	13^c^ (46.4)	11 (44.0)	
Infectious	6	1 (3.6)	5 (20.0)	
Genetic	3^c^	2^c^ (7.1)	1 (4.0)	
Immune	2	2 (7.1)	0	
Unknown	20 (39.6)	12 (42.9)	8 (32.0)	
**Current ASM use; *n* (%)**				0.779^b^
1 ASM	3 (5.7)	0	3 (12.0)	
2 ASMs	16 (30.2)	10 (35.7)	6 (24.0)	
3 ASMs	30 (56.6)	16 (57.1)	14 (56.0)	
4 ASMs	4 (7.5)	2 (7.1 %)	2 (8.0)	
**ASM with TPM/ZNS; *n* (%)**				0.907^b^
2–4 ASMs with TPM or ZNS	20 (40.0)	11 (39.3)	9 (40.9)	
2–4 ASMs without TPM or ZNS	30 (60.0)	17 (60.7)	13 (59.1)	

*Note*: Epilepsy duration was not available for one patient.

Abbreviations: ASM, anti‐seizure medication; TLE, temporal lobe epilepsy; TPM, topiramate; XLE, extra‐temporal epilepsy; ZNS, zonisamide.

^a^ Mann–Whitney *U* test.

^b^ Pearson's chi‐squared test.

^c^ Two patients with tuberosis sclerosis were included in both structural and genetic etiologies.

All patients were treated with ASMs, ranging from 1 to 4. ASM use was categorized into two main groups: those taking 1–2 ASMs and those taking 3–4 ASMs. Among newer generation ASMs, treatment with topiramate (TPM) or zonisamide (ZNS) has been specifically associated with impaired cognitive performance (Lähde et al. 2021; Witt et al. 2015). To evaluate the impact of TPM and ZNS on cognitive functions, patients were further divided into two subgroups: those taking 2–4 ASMs including TPM or ZNS, and those taking 2–4 ASMs without these medications. Three patients using only one ASM were excluded from this analysis. The study did not include any patients on combination therapy with both TPM and ZNS, nor those on monotherapy with either TPM or ZNS.

### 2.4 Neuropsychological Evaluation

The neuropsychological examination was conducted as part of the assessment for neurostimulation therapy, with the evaluation closest to implantation included in the study. Forty‐eight patients were evaluated prior to implantation, and five ANT‐DBS patients were evaluated 15–20 months after implantation.

The cognitive domains evaluated were (1) utfAEFs; (2) ML; (3) LFs; and (4) VFs. AEFs were assessed using the Controlled Oral Word Association Test (COWAT), including semantic (animals) and phonemic (letters P‐A‐S; Finnish version of F‐A‐S) fluency (Benton and Hamsher 1976), the Trail Making Test A & B (TMT A + B) (Army Individual Test Battery 1944), the Digit Span subtest of the Wechsler Adult Intelligence Scale III (WAIS‐III) (Wechsler 1955), and the Stroop Color‐Word Interference Test (Stroop 1935). ML were evaluated using the Rey Auditory Verbal Learning Test (RAVLT) (Rey 1964) and the Drawing from Memory subtest of the Rey–Osterrieth Complex Figure Test (ROCFT). The RAVLT analysis included the total score (total number of words recalled in trials 1 through 5), post‐interference recall (number of words recalled after interference), and recognition (number of correctly recognized words in delayed recall). LFs were assessed using the similarities subtest of the WAIS‐III (Wechsler 1955), whereas VFs were evaluated using the Copying and Copying Time subtests of the ROCFT (Osterrieth 1944) and the Block Design subtest of the WAIS‐III.

Patients were assessed as part of the clinical protocol, and the neuropsychological test battery was tailored to each patient's clinical situation. Consequently, not all tests were administered to every patient. On average, 5.4 tests (range: 2–6) were conducted for AEFs, 5.4 tests (range: 1–6) for ML, 0.9 tests (range: 0–1) for LFs, and 1.7 tests (range: 1–2) for VFs. Two patients did not undergo any LF tests.

Raw scores from the cognitive tests were converted into age‐standardized *Z*‐scores using published Finnish (Wechsler 1955) and international (Mitrushima et al. 2005) normative data. The scores were adjusted so that higher values consistently indicated better performance. To derive a composite score for each cognitive domain, the average of the *Z*‐scores was calculated. In this study, *Z*‐scores were categorized as follows: normal performance for *Z*‐scores > −1, mild impairment for −2 < *Z*‐scores ≤ −1, moderate impairment for −3 < *Z*‐scores ≤ −2, and severe impairment for *Z*‐scores ≤ −3.

### 2.5 Statistical Analysis

The normality of distribution was tested using the Shapiro–Wilk test and by examining kurtosis and skewness values. Descriptive data (frequencies and proportions, or median and range) were reported to summarize patients’ demographics and clinical characteristics. Due to the positively skewed distribution of continuous variables, nonparametric tests—the Mann–Whitney *U* test or the Kruskal–Wallis test—were applied to compare differences between study groups. The severity of impairment, as defined by *Z*‐scores in each cognitive domain, was analyzed using Friedman's two‐way analysis of variance. Pearson's chi‐squared test was used to evaluate associations between groups and categorical variables, provided that assumptions were met; otherwise, Fisher's exact test was applied. Statistical significance was set at *p* < 0.05, and all analyses were performed using IBM SPSS Statistics version 24.0.

## 3 Results

### 3.1 Performance in Different Cognitive Domains

**FIGURE 1:**
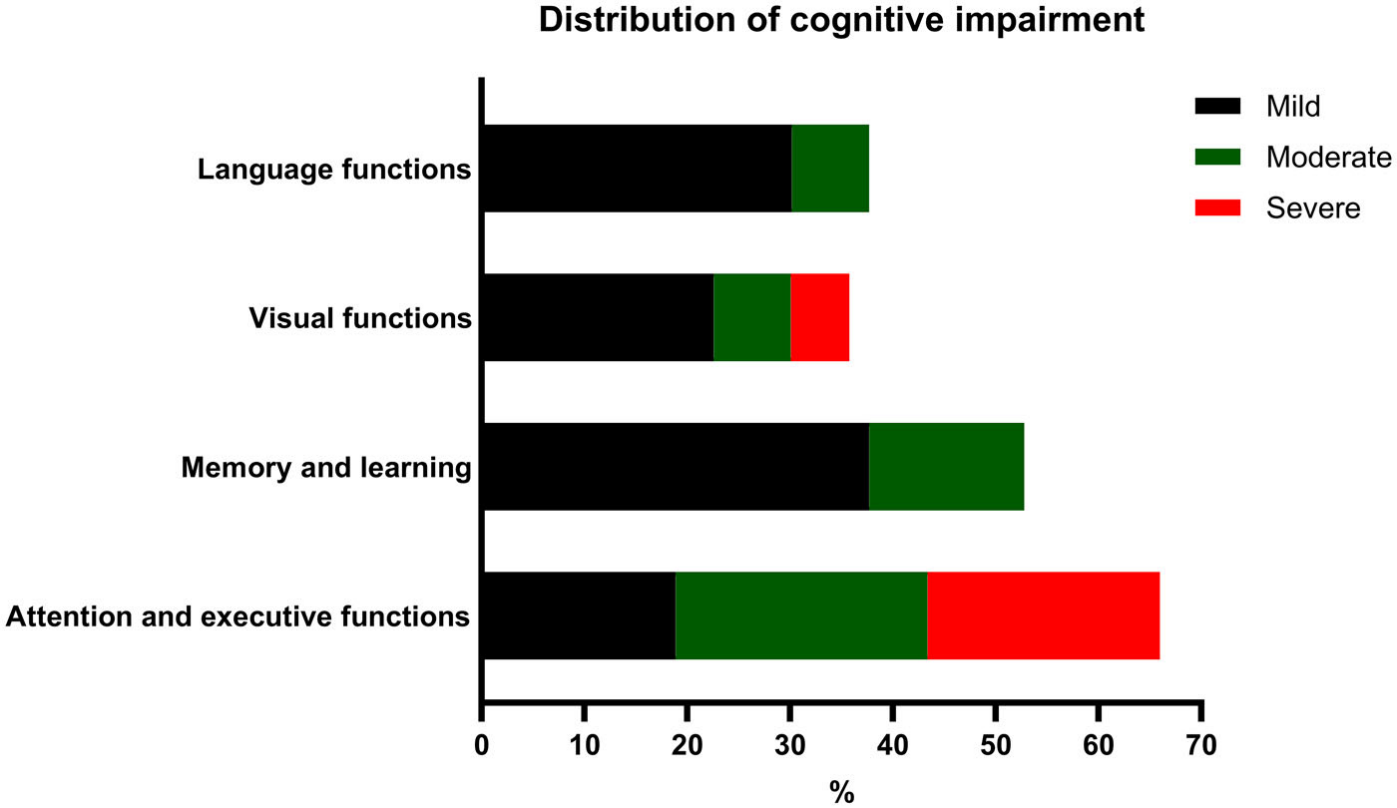
**Cognitive impairment and severity across the four domains in the whole study group (*N* = 53)**. Normal performance is defined by Z‐scores > −1, mild impairment −2 < Z‐scores ≤ −1, moderate impairment −3 < Z‐scores ≤ −2, and severe impairment Z‐scores ≤ −3.

AEFs were impaired in 66.0% (*n* = 35) of patients, with the majority exhibiting either moderate (24.5%, *n* = 13) or severe (22.6%, *n* = 12) impairment, and 18.9% (*n* = 10) showing mild impairment. ML deficits were observed in 52.8% (*n* = 28) of patients, primarily with mild (37.7%, *n* = 20) or moderate (15.1%, *n* = 8) impairment; none experienced severe impairment. LFs were impaired in 39.2% (*n* = 20) of patients, with 31.4% (*n* = 16) showing mild impairment and 7.8% (*n* = 4) moderate impairment, whereas no severe impairment was noted. VFs were impaired in 35.8% (*n* = 19) of patients, including 22.6% (*n* = 12) with mild, 7.5% (*n* = 4) with moderate, and 5.7% (*n* = 3) with severe impairment (Figure 1).

**FIGURE 2:**
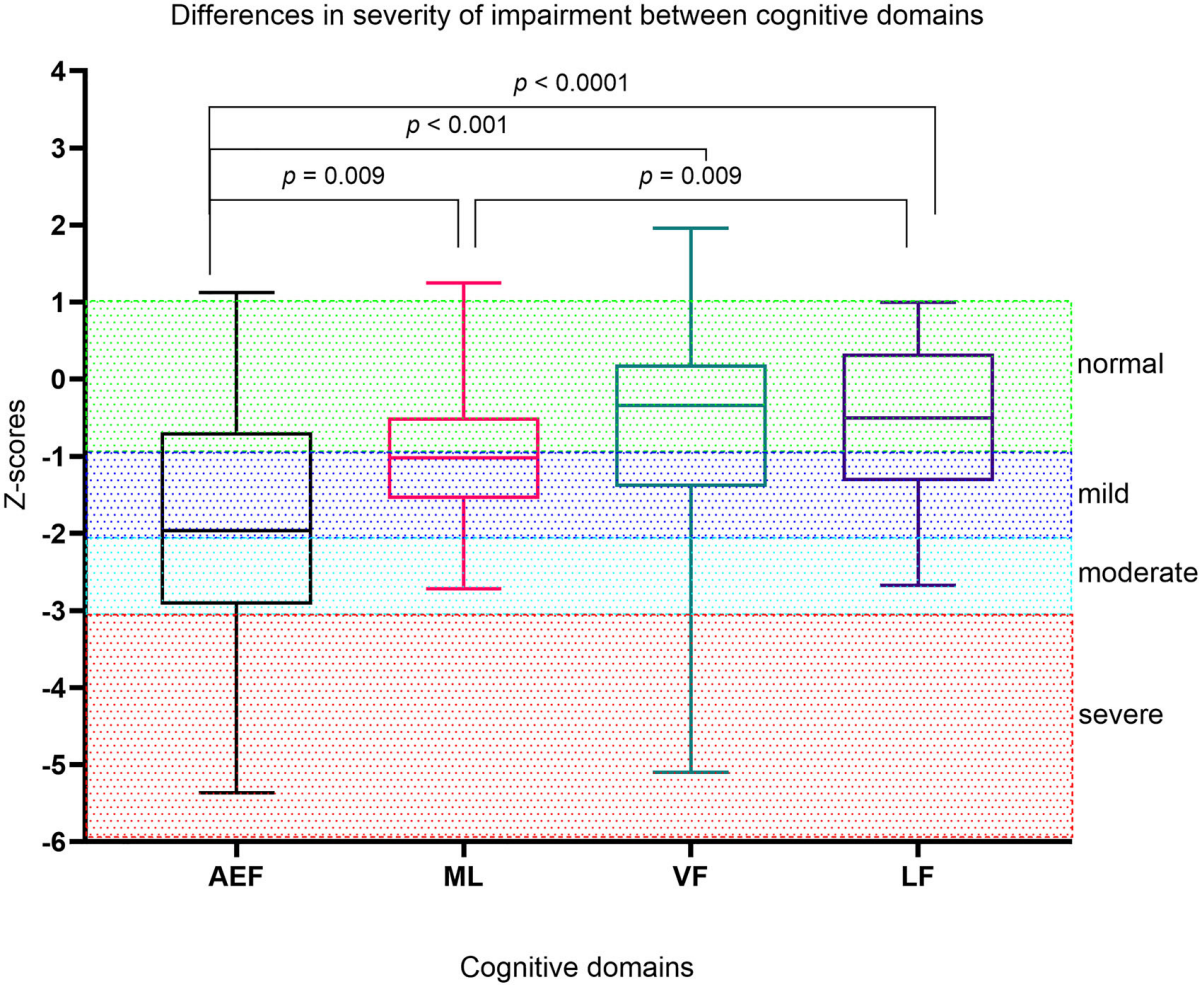
Box plots with median Z‐scores (range) among the cognitive domains. Friedman's two‐way analysis. Because multiple tests were performed for background measurements, we applied Bonferroni correction to control for multiple comparisons; thus, *p* < 0.017 was considered statistically significant. AEF, attention and executive functions; LF, language functions; ML, memory and learning; VF, visual functions.

When comparing cognitive performance across the four domains, AEFs were significantly more impaired than ML (*p* = 0.009), VFs (*p* < 0.001), and LFs (*p* < 0.001) (Figure 2). Although ML showed greater impairment compared to visual and LFs, a significant difference was noted only in comparison to LFs (*p* = 0.009). VFs did not differ significantly from ML (*p* = 0.222) or LFs (*p* = 0.981).

### 3.2 Differences in Cognitive Performance Between Patients Intended for VNS and ANT‐DBS Therapy

**FIGURE 3:**
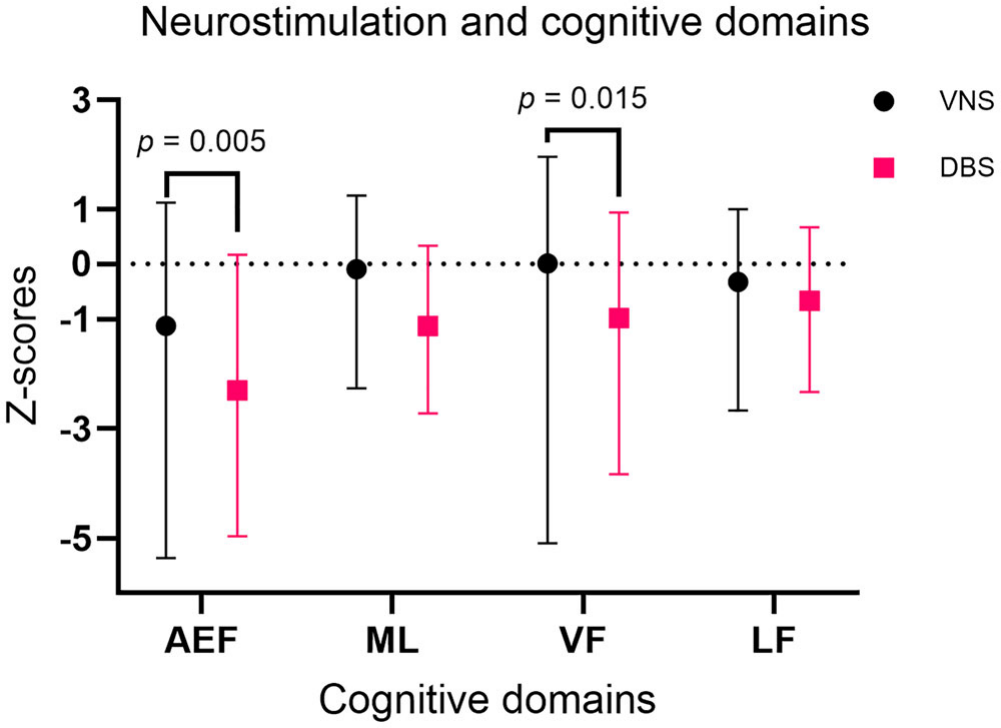
Bar graph with median (range) *Z*‐scores for cognitive domains in patients intended for treatment with VNS and ANT‐DBS. Normal performance is defined by Z‐scores > −1, mild impairment −2 < Z‐scores ≤ −1, moderate impairment −3 < Z‐scores ≤ −2, and severe impairment Z‐scores ≤ −3. AEF, attention and executive functions; LF, language functions; ML, memory and learning; VF, visual functions; VNS, vagus nerve stimulation.

Among the study population, 52.8% (*n* = 28) of patients were considered for VNS treatment, and 47.2% (*n* = 25) for ANT‐DBS treatment. Analysis based on *Z*‐scores revealed that patients intended for ANT‐DBS treatment exhibited significantly greater impairments in AEFs, as well as in VFs, compared to those considered for VNS treatment (*p* = 0.005 and *p* = 0.015, respectively). No significant differences were observed in the severity of impairments across the other cognitive domains (Figure 3).

Of the 25 patients considered for ANT‐DBS treatment, eight (32%) had a prior history of unsuccessful VNS therapy. We compared the baseline cognitive performance between these eight patients and the remaining 17 ANT‐DBS patients without previous VNS. The results indicated no significant differences in the severity of impairments across all cognitive domains (*p* > 0.05). Notably, none of the patients considered for VNS treatment had a prior history of ANT‐DBS therapy.

### 3.3 Impact of Clinical Characteristics on Performance in Cognitive Domains

We assessed the effects of various clinical characteristics on cognitive performance. The level of education had a notable impact on cognitive outcomes. Patients with more than 12 years of education exhibited significantly higher median *Z*‐scores in AEFs, LFs, and VFs compared to patients with fewer years of education (Table 2).

**TABLE 2:** Impact of clinical characteristics on performance in cognitive domains.

Characteristics	Attention and executive functions		Memory and learning		Visual function		Language function	
	Median (range) *Z* scores	*p* value	Median (range) *Z* scores	*p* value	Median (range) *Z* scores	*p* value	Median (range) *Z* scores	*p* value
Gender		0.676^a^		0.899^a^		0.393^a^		0.166^a^
Female (*n* = 21)	−1.99 (−5.36 to 0.23)		−1.02 (−2.67 to 1.15)		−0.83 (−2.91 to 1.96)		−0.84 (−2.67 to 0.67)	
Male (*n* = 32)	−1.94 (−4.96 to 1.12)		−0.97 (−2.72 to 1.25)		−0.08 (−5.10 to 1.17)		−0.33 (−2.33 to 1.00)	
Level of education		**0.030** ^b^		0.965^b^		**0.023** ^b^		**0.009** ^b^
9 years (*n* = 6)	−2.10 (−2.50 to −0.68)		−1.09 (−1.86 to −0.28)		−0.53 (−1.93 to 0.34)		−0.67 (−2.67 to 0.67)	
10–12 years (*n* = 36)	−2.19 (−11.55 to 1.12)		−1.02 (−2.72 to 1.25)		−0.69 (−5.10 to 0.94)		−0.67 (−2.33 to 1.00)	
>12 years (*n* = 11)	−0.82 (−4.14 to 0.58)		−1.03 (−2.33 to 0.77)		0.07 (−1.54 to 1.96)		0.00 (−0.67 to 1.00)	
Age groups		0.125^b^		0.406^b^		0.976^b^		0.092^b^
18–29 years (*n* = 16)	−2.06 (−4.96 to 0.23)		−1.05 (−2.13 to 1.14)		−0.39 (−2.29 to 0.94)		−0.33 (−2.00 to 0.67)	
30–49 years (*n* = 29)	−2.05 (−5.36 to 0.58)		−0.92 (−2.71 to 0.77)		−0.34 (−5.10 to 1.17)		−0.67 (−2.67 to 1.00)	
50–70 years (*n* = 8)	−0.67 (−4.40 to 1.12)		−1.40 (−2.67 to 1.25)		−0.19 (−3.83 to 1.96)		0.50 (−1.67 to 1.00)	
Epilepsy types		0.483^b^		0.317^b^		0.282^b^		0.660^b^
Temporal lobe (*n* = 24)	−1.9 (−4.8 to 0.31)		−0.85 (−2.67 to 1.25)		−0.15 (−5.1 to 0.94)		−0.5 (−2.67 to 1.00)	
Extratemporal (*n* = 19)	−1.40 (−4.96 to 0.58)		−1.3 (−2.33 to 1.96)		−0.52 (−2.29 to 1.9)		−0.33 (−2.33 to 1.00)	
Multifocal (*n* = 10)	−2.74 (−5.38 to 1.12)		−1.01 (−2.72 to 0.33)		−1.04 (−3.83 to 0.08)		−1.33 (−2.33 to 0.67)	
Epilepsy duration		0.400^b^		0.624^b^		0.409^b^		**0.035** ^b^
<10 years (*n* = 17)	−1.59 (−4.79 to 0.24)		−1.05 (−2.05 to 1.15)		−0.09 (−5.1 to 1.96)		−0.33 (−2.33 to 0.67)	
10–20 years (*n* = 15)	−1.46 (−4.12 to 1.12)		−0.83 (−2.33 to 0.70)		0.01 (−2.29 to 1.17)		−0.33 (−1.67 to 1.00)	
>20 years (*n* = 21)	−2.31 (−5.36 to 0.58)		−1.1 (−2.72 to 1.25)		−0.67 (−4.66 to 0.94)		−1.00 (−2.67 to 1.00)	

*Note*: Normal performance is defined by Z‐scores > −1, mild impairment −2 < Z‐scores ≤ −1, moderate impairment −3 < Z‐scores ≤ −2, and severe impairment Z‐scores ≤ −3. Bold values represent significant *P*‐values.

^a^ Mann–Whitney *U* test.

^b^Kruskal–Wallis test.

Regarding epilepsy‐related factors, LFs were more impaired in patients with an epilepsy duration of over 20 years compared to those with a duration of less than 10 years (−1.00 vs. −0.33, *p* = 0.035). Age, gender, and epilepsy type did not significantly affect cognitive performance.

### 3.4 Impact of ASM Treatment on Performance in Cognitive Domains

**FIGURE 4:**
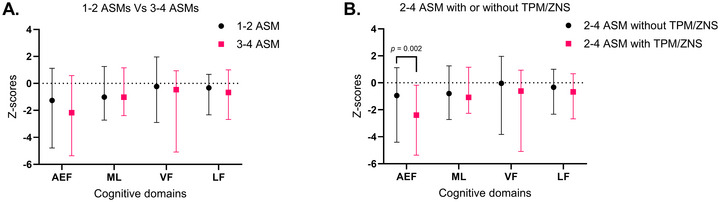
Median (range) *Z*‐scores for cognitive domains in patients treated with (A) 1–2 ASMs versus 3–4 ASMs and (B) 2–4 ASMs with TPM/ZNS versus 2–4 ASMs without TPM/ZNS. AEF, attention and executive functions; ASM, anti‐seizure medication; LF, language functions; ML, memory and learning; TPM, topiramate; VF, visual functions; ZNS, zonisamide.

The effects of ASMs on cognitive performance were evaluated first by comparing the total number of concomitant ASMs (patients with 1–2 ASMs vs. patients with 3–4 ASMs) and second by comparing patients with or without TPM and/or ZNS. The total number of ASMs did not show a statistically significant effect on cognitive performance (Figure 4A). However, patients taking TPM/ZNS in combination with 2–4 ASMs showed significantly greater impairment in AEFs than those taking 2–4 ASMs without TPM/ZNS (*p* = 0.002, Figure 4B).

## 4 Discussion

This study highlights several key aspects of cognition in DRE. First, impairments across all four cognitive domains were identified in more than one‐third of patients evaluated for neurostimulation treatment, whether undergoing VNS or ANT‐DBS. These deficits were not uniformly distributed: Approximately two‐thirds of patients exhibited impairments in AEFs, half in ML, and only about one‐third in visual and LFs. Notably, the impairments in AEFs were significantly more pronounced and severe than those in other domains. Additionally, the ASM regimen was associated with impairments in executive functions but not in other cognitive domains. Finally, cognitive profiles differed between patients selected for VNS and ANT‐DBS, with those considered for ANT‐DBS showing greater impairments in AEFs, as well as in VFs.

A neuropsychological evaluation is essential prior to both resective surgery and the initiation of neurostimulation therapy, due to the cognitive challenges associated with DRE. In this study, patient inclusion was based on the decision to initiate neurostimulation treatment with either VNS or ANT‐DBS, often involving patients with severe DRE and cognitive impairments. Consequently, over one‐third of our cohort exhibited deficits across all cognitive domains, with AEFs being the most affected. These findings are consistent with previous research indicating that deficits in attention, executive functions, and memory are prevalent cognitive impairments in adults with epilepsy (Elger et al. 2004; Novak et al. 2022). A prior study reported that deficits in AEFs were present in up to 75% of DRE patients (Lähde et al. 2021).

Among the static factors evaluated in this study, education level significantly influenced cognitive performance. Patients with more than 12 years of education performed markedly better in AEFs, as well as in visual and LFs. This finding aligns with evidence that factors such as low educational level contribute to cognitive deficits in epilepsy patients (Peltola et al. 2024). In our previous study, more than 80% of DRE patients who had completed only primary education (9 years) were found to be severely impaired in AEFs as measured with the EpiTrack test (Lähde et al. 2021). Our current findings add another layer to this observation by highlighting an association between specific cognitive domains and low educational level in DRE patients. Other clinical factors, such as age, gender, or duration and type of epilepsy, had no effect on cognitive performance.

Several dynamic factors can influence cognitive functioning in patients with DRE, including seizure frequency and severity, interictal epileptiform discharges, psychiatric comorbidities, and treatment‐related factors (Elger et al. 2004; Kwan and Brodie 2001). Among these, a high number of concomitant ASMs and specific ASMs are often linked to reduced cognitive performance (Witt et al. 2013, 2015; Witt and Helmstaedter 2013). In this study, the number of ASMs did not correlate with cognitive performance. However, the use of 2–4 ASMs, including TPM or ZNS, impaired AEFs without affecting other domains. Similarly, a retrospective study of 135 epilepsy patients showed that adjunctive ZNS and TPM worsened performance in executive function tests but did not impact verbal memory (Meschede et al. 2020). Evidence varies for each ASM, and research is ongoing regarding the effects of newer ASMs on mood, behavior, and cognition. Selecting ASMs for patients with DRE is crucial, as some ASMs negatively impact cognition, tolerability, compliance, and long‐term treatment retention (Witt et al. 2015).

VNS and ANT‐DBS may have both direct and indirect positive effects on cognition (Chan et al. 2018; Mertens et al. 2022; Sun et al. 2017). Indirect effects result from decreased seizure frequency, a reduced number of administered ASMs, potential improvements in mood, and fewer interictal epileptiform discharges. We recently observed significant improvement in AEFs among DRE patients receiving VNS therapy over a 5‐year follow‐up period (Lähde et al. 2024). Similarly, a long‐term extension of the SANTE trial reported significant improvements in AEFs in patients undergoing ANT‐DBS over 5 years (Salanova et al. 2015). Consequently, neurostimulation may offer a more favorable cognitive side‐effect profile compared to typical ASM treatment. Therefore, evaluating cognitive performance—especially executive functions—should be integral to assessing treatment progress in patients receiving neurostimulation therapy. Conducting repeated neuropsychological examinations is impractical in a clinical setting, so employing a feasible, convenient, and reliable cognitive screening tool is essential for optimizing the cognitive benefits of neurostimulation. Given that AEFs were the most compromised domains in our study, and previous research (Lähde et al. 2024; Salanova et al. 2015) suggests these functions may improve with neurostimulation, it is highly recommended to assess these domains using a tool like EpiTrack (Helmstaedter and Witt 2012) or EpiTrack Plus (Witt et al. 2015), which also includes a test of verbal memory.

Our study identified distinct differences in the cognitive profiles of patients undergoing evaluation for VNS and ANT‐DBS. Patients considered for ANT‐DBS treatment were significantly more impaired in AEFs compared to those considered for VNS. This poorer performance may be due to ANT‐DBS candidates being among the most challenging to treat, often with more severe epilepsy. Interestingly, cognitive profiles may influence ANT‐DBS treatment outcomes, with previous research suggesting that better performance in AEFs predicts favorable clinical outcomes post‐ANT‐DBS (Järvenpää et al. 2018). Additionally, patients intended for DBS implantation showed poorer performance in VFs than those intended for VNS, further highlighting the impact of executive functions on outcomes, as tests within the VF domain (ROCFT and block design) also require executive functioning. At present, neuropsychological evaluation can provide additional support in determining whether VNS or ANT‐DBS is the more appropriate treatment option for a given patient.

One of the main limitations of this study was the small sample size. Conducting the research within a single‐center setting contributed to this limitation, making it challenging to attain statistical significance, particularly in subgroup analyses. Additionally, although most patients underwent neuropsychological examination prior to implantation, five were evaluated postimplantation, which may have influenced the results. However, all postimplantation evaluations were conducted within 2 years of surgery, whereas cognitive improvements associated with neurostimulation typically do not manifest until at least 2 years after therapy initiation (Lähde et al. 2024). Furthermore, the number of neuropsychological tests varied across domains, and not all tests were used in every evaluation. VFs were assessed with only two tests, and LFs with one, whereas AEFs were evaluated with six tests. Lastly, the absence of systematic postoperative neuropsychological assessments represents a limitation of this study. Future investigations incorporating longitudinal cognitive evaluations would be valuable to examine treatment‐related changes and their relationship to baseline performance profiles.

## 5 Conclusions

There is an increasing focus on the neuropsychological aspects of DRE. Our study underscores the importance of assessing cognitive function before initiating neurostimulation treatment in this patient population. Specifically, the baseline neuropsychological profile may help guide the selection between these two neurostimulation therapies. Better AEFs have been associated with more favorable outcomes following ANT‐DBS therapy, whereas DRE patients with impairments in these domains may experience significant cognitive benefits from VNS treatment. Furthermore, given that neurostimulation may influence cognitive function through both direct and indirect mechanisms, cognitive evaluation should also be an integral part of treatment monitoring.

## Author Contributions

**Lähde Niina**: study concept and design, acquisition of data, analysis and interpretation, drafting the manuscript. **Basnyat Pabitra**: acquisition of data, statistical analyses and interpretation, and revised manuscript. **Lehtinen Hanna**: data analysis and interpretation, revised manuscript, and statistical analyses. **Rosti‐Otajärvi Eija**: acquisition of data, analysis and interpretation, revised manuscript. **Kämppi Leena**: analysis and interpretation, revised manuscript. **Peltola Jukka**: study concept and design, critical revision, study supervision. All authors have read and approved the final manuscript.

## Funding

The authors have nothing to report.

## Ethics Statement

This was a non‐interventional study in which data was collected prospectively but analyzed retrospectively from a VNS quality register at Tampere University Hospital, therefore not requiring ethics committee approval according to Finnish Law on Research.

## Conflicts of Interest

Lähde Niina has participated in a clinical trial for UCB; received speaker´s honoraria from LivaNova (OmaMedical). Kämppi Leena has received honoraria from UCB, Angelini Pharma and Sobi, congress support from UCB and Angelini Pharma, and research funding from the Academy of Finland, Finnish Cultural Foundation and HUS Neurocenter. Peltola Jukka has participated in clinical trials for Eisai, UCB, and Bial; received research grants from Angelini Pharma, Eisai, Medtronic, UCB, and LivaNova; received speaker´s honoraria from LivaNova, Angelini Pharma, Eisai, Jazz Pharma, Medtronic, Orion Pharma, and UCB; received support for travel to congresses from LivaNova, Eisai, Medtronic, and UCB; and participated in advisory boards for LivaNova, Angelini Pharma, Jazz Pharma, Eisai, Medtronic, UCB, and Pfizer. The remaining authors have no conflicts of interest.

## Data Availability

The data that support the findings of this study are available upon request from the corresponding author. The data are not publicly available due to privacy or ethical restrictions.
